# Pseudo-Darwinian evolution of physical flows in complex networks

**DOI:** 10.1038/s41598-020-72379-8

**Published:** 2020-09-23

**Authors:** Geoffroy Berthelot, Liubov Tupikina, Min-Yeong Kang, Bernard Sapoval, Denis S. Grebenkov

**Affiliations:** 1 Centre de Mathématiques Appliquées, CNRS – Ecole Polytechnique, IP Paris, 91128 Palaiseau, France; 2 Research Laboratory for Interdisciplinary Studies (RELAIS), 75012 Paris, France; 3 Institut National du Sport, de l’Expertise et de la Performance (INSEP), 75012 Paris, France; 4 Center for Research and Interdisciplinarity (CRI), Université de Paris – INSERM (U1284), 75004 Paris, France; 5 Bell Labs Nokia, Paris , France; 6 Laboratoire de Physique de la Matière Condensée (UMR 7643), CNRS – Ecole Polytechnique, IP Paris, 91128 Palaiseau, France

**Keywords:** Complex networks, Statistical physics, Permeation and transport

## Abstract

The evolution of complex transport networks is investigated under three strategies of link removal: random, intentional attack and “Pseudo-Darwinian” strategy. At each evolution step and regarding the selected strategy, one removes either a randomly chosen link, or the link carrying the strongest flux, or the link with the weakest flux, respectively. We study how the network structure and the total flux between randomly chosen source and drain nodes evolve. We discover a universal power-law decrease of the total flux, followed by an abrupt transport collapse. The time of collapse is shown to be determined by the average number of links per node in the initial network, highlighting the importance of this network property for ensuring safe and robust transport against random failures, intentional attacks and maintenance cost optimizations.

## Introduction

Transport in complex systems can describe a variety of natural and human-engineered processes including biological^1,2^, societal and technological ones^3,4^. Common examples include blood vessel network and the lung airway tree^5^ that deliver blood and oxygen molecules, respectively; braided streams, consisting in a network of water channels, that occur in rivers and in glaciated landscapes when the discharge of water cannot transport its load or when sediment is deposited on the floor of the channel^6,7^; transportation networks for passengers^8–10^; social networks, in which the social and experience flow is progressively formed between individuals over time. These empirical networks are often scale-free and characterized by a degree distribution that follows a power law $P(k) \sim k^{-\gamma }$ with an exponent $\gamma$ often in a range between 2 and 3 or a truncated power law^11,12^. The morphological organization of transport in static scale-free networks was investigated from various perspectives^13–16^. It is known that transport through scale-free networks and their functionality in general are vulnerable to the intentional attack to a few vertices with high degree, but remain very robust to random failures^17,18^. While the percolation by deleting *nodes* has been extensively studied^15,19,20^, the effect of progressive *link* removal on transport in scale-free networks is not well understood. In this paper we aim at investigating how the temporal evolution of local connectivities can lead to emergence of ordered structures in scale-free networks under different strategies of link removal. While we will focus on physical transport systems, where a flux is an electric current or the quantity of transferred materials or molecules over time, the obtained results are of much broader scope and reveal the fundamental principles of structural evolution of general transport networks.

## Methods

We construct a random scale-free network on an $n \times n$ square lattice. Its links are generated by using the uncorrelated configuration model^21^ with a given degree exponent $\gamma$. We consider the resistance $r_{i,j}$ of each link as a function of the Euclidean distance $d_{i,j}$ between the nodes *i* and *j*: $r_{i,j} =d_{i,j}^{\beta }$, with an exponent $\beta$. In an electric or hydraulic circuit, the resistance of a wire or a tube is proportional to its length, and $\beta =1$. In turn, most former studies on transport in resistor networks supposed constant link resistance, i.e., $\beta =0$. In each random realization of the resistor network with prescribed exponents $\gamma$ and $\beta$, we select randomly a source and a drain nodes, at which the potential is fixed to be 1 and 0, respectively. We ensure the distance (in terms of the number of nodes) between the source and drain is $\ge 4$. The system of linear Kirchhoff’s equations^22^ for the potential on other nodes is solved numerically using a custom routine in Matlab. Then the distributions of nodes potentials and currents in links are obtained. Such a point-to-point transport was shown to be self-organized into two tree-like structures, one emerging from the source and the other converging to the drain^16^. These trees merge into a large cluster of the remaining nodes that is found to be quasi-equipotential and thus presents almost no resistance to transport.

We consider three dynamics of network evolution, in which links are progressively removed according to one of the following strategies: at each step, one removes (i) randomly chosen link, (ii) the link with the strongest flux, and (iii) the link with the weakest flux. These three evolution strategies are meant to model respectively (i) progressive failures in a system in random (unrelated) places (e.g., due to material aging); (ii) intentional attacks on the network by removing the most relevant links; and (iii) a kind of progressive optimization of the system by removing least used elements. While the first two evolution strategies have been earlier studied (but mainly for nodes removal)^23^, we are not aware of former works on the latter network evolution that we call “Pseudo-Darwinian strategy” of network percolation. Such a strategy is often employed in nature, e.g., as the mechanism of capillary network remodeling during morphogenesis^24^.

At each evolution step, we first solve the system of Kirchhoff’s equations to calculate fluxes in all links, and then we remove a link according to the selected strategy. After link removal, we also remove “dead-ends” (i.e., nodes that have a single link), thus imposing that any existing node after an evolutionary step has at least two links. We keep track of the *total flux* *Q*, i.e., the flux that enters into the network from the source node. As we investigate the evolution of the network after successive link removals, it is natural to associate the number of evolution steps with “time” *t*, with 0 being the initial time, before the evolution takes place. We denote $Q_0$, $L_0$ and $N_0 = n^2$ as the total flux, the number of links, and the number of nodes of the initial network, respectively. The evolution ends when at least one of the following conditions is met: (i) no path exists between the source and the drain (i.e. the source and drain are disconnected), (ii) the source or the drain is removed from the network, (iii) a portion of the network—a subgraph containing more than one node—is disconnected from the rest of the network containing the source and drain. We call this moment as “time of collapse” $t_c$: as one of the previous conditions is met, transport is no longer maintained through the whole network.

**Figure 1:**
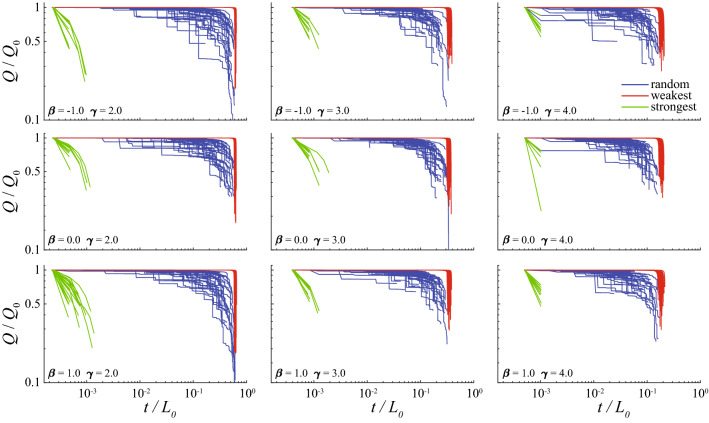
Evolution of the total flux *Q* (rescaled by $Q_0$) for a scale-free network with $N_0 = 40\times 40$ nodes for nine combinations of parameters $\gamma$ and $\beta$: $\gamma = \{2,3,4\}$ (left, middle and right columns) and $\beta = \{-1,0,1\}$ (top, middle and bottom rows). In each plot, 60 curves correspond to 60 random realizations of the initial network.

## Results

**Figure 2:**
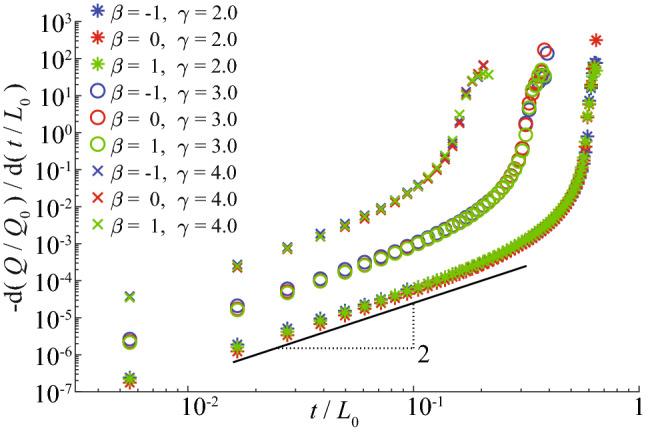
Time derivative of the total flux for a scale-free network with $N_0 = 40\times 40$ nodes and different values of $\gamma$ and $\beta$ for the pseudo-Darwinian strategy only. The slope is identical for all values of $\gamma$ and $\beta$, suggesting a universal phenomenon. The derivative here is calculated as an average from 60 realizations.

**Figure 3:**
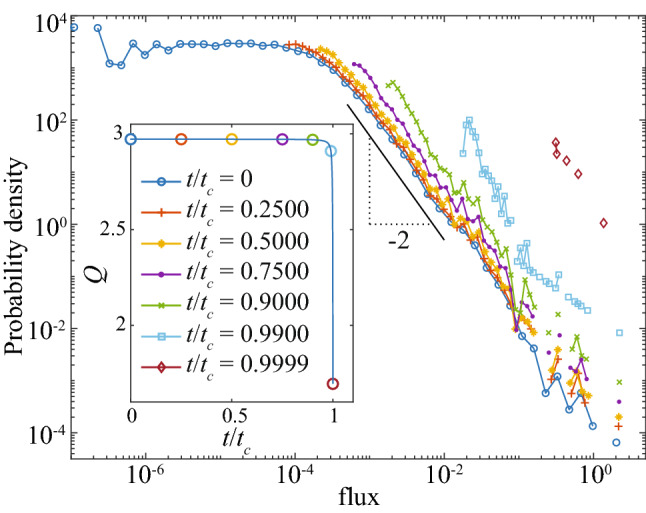
The flux probability density at selected moments of $t/t_c$, for a network with $N_0 = 100 \times 100$ nodes, $\gamma = 2.5$, $\beta = 1$, and $L_0 = 21,040$ initial links. Corresponding total fluxes are shown in the inset.

**Figure 4:**
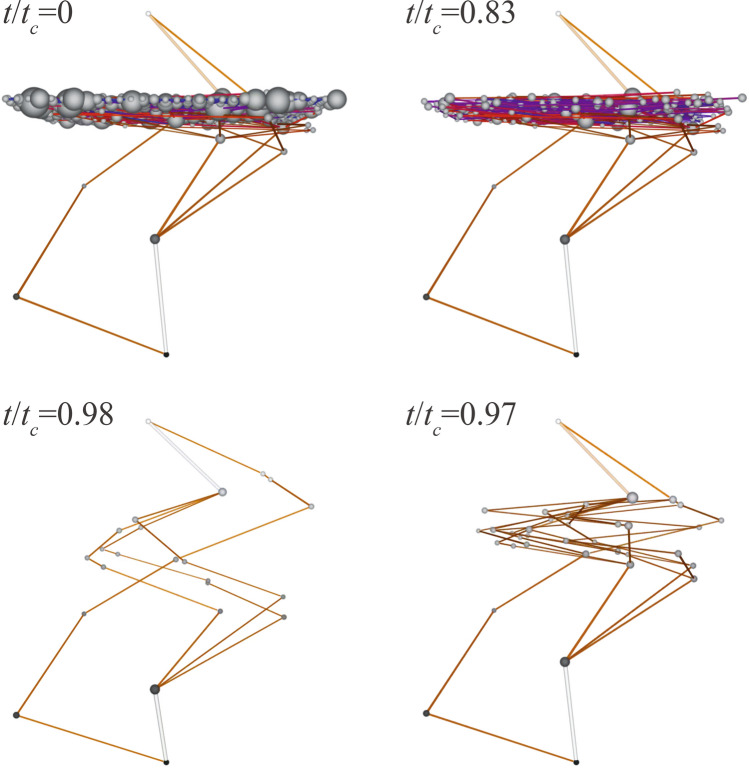
Visualization of evolution of a scale-free network (with $N_0 =30\times 30$ nodes, $\gamma =2.5$ and $\beta =1$; A video is available online). Each node of the network is shown by a ball whose radius is proportional to the square root of its connectivity. The planar coordinates of the balls are the positions of the corresponding nodes on the square lattice, whereas the height *Z* represents the potential at the node by a linear relation $Z=V$. Each link brightness is proportional to the magnitude of its current (in addition, blue colors are used for very small currents). The quasi-equipotential cluster is qualitatively identified as a large ensemble of nodes almost at the same potential. Four panels represent the network at four moments, $t/t_c$, during evolution (clock-wise direction): 0 (initial state), 0.83, 0.97, and 0.98. Along the evolution process, the weakest links are removed successively. Note that links in the cluster are removed during the majority of evolution period.

We first explore a scale-free resistor network with $N_0 =40\times 40$ nodes and parameters $\gamma \in \{2, 3, 4\}$ and $\beta \in \{-1,0,1\}$. Figure 1 shows how the total flux *Q*, rescaled by its initial value $Q_0$, evolves with time *t* for different networks ($\gamma$, $\beta$) and strategies. Expectedly, the progressive removal of the strongest links leads to a very rapid transportation collapse, whereas this decline is the slowest for the pseudo-Darwinian strategy. To better understand the effect of this strategy, we also calculate the derivative of the total flux *Q* with respect to time (Fig. 2). Two regimes are observed: a slow, power law decay, followed by an abrupt decay. In the first regime, we find the universal scaling exponent 2 of the derivative of the total flux for all $\gamma$ and $\beta$ values, implying

1 $$\begin{aligned} Q(t)/Q_0 \simeq 1 - C (t/t_c)^3 \qquad (t \ll t_c), \end{aligned}$$

where *C* is a nonuniversal constant. The distribution of fluxes in links along the evolution process (Fig. 3) shows that the removal of the weakest link at each evolution step does not affect the distribution of large fluxes for the most period of evolution. For this period, the total flux remains almost the same. Figure 4 helps to understand this robustness: the removal occurs mostly in the links in the quasi-equipotential cluster where connections are abundant while the links with large currents are kept.

**Figure 5:**
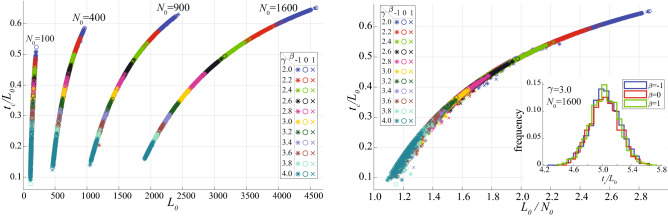
Rescaled time of collapse $t_c / L_0$ versus the initial number of links $L_0$**(left)** or versus the average initial number of links per node $L_0 / N_0$**(right)** for 4 different network sizes ($N_0=100$, $N_0=400$, $N_0=900$, $N_0=1600$), 11 values of $\gamma$ (evenly spaced from 2 to 4) and 3 values of $\beta$ ($\beta =\{-1,0,1\}$). Each colored marker represents the time of collapse obtained from 200 realizations for each triplet of $\gamma$ (specified by color), $\beta$ (specified by marker) and network size $N_0$. The parameter $\beta$ has no significant effect on $t_c$, as confirmed in the inset, where three colored lines show the empirical distributions of $t_c$ for three different values of $\beta$ (obtained from 2,000 simulations).

The time of collapse $t_c$ is related to the degree exponent $\gamma$ in all strategies: networks with lower $\gamma$ values produce higher $t_c$ (Fig. 1). This highlights the role of connectivity in resisting against random or targeted attacks. This effect is illustrated for the pseudo-Darwinian evolution by plotting the time of collapse rescaled by $L_0$, $t_c / L_0$, versus the initial number of links $L_0$ for various network sizes (Fig. 5(left)). Further rescaling the horizontal axis $L_0$ by the initial number of nodes, $N_0$, results in a collapse of all curves into a single master curve that determines $t_c/L_0$ as a function of the initial average degree, $L_0/N_0$, independently of the network structure ($\gamma$, $\beta$) and size $N_0$ (Fig. 5(right)). Thus we conclude that higher average degree in an initial network helps in delaying the time $t_c$ of transport collapse.

## Discussion

In our setup, the total flux describes a network functionality and its ability to transport or distribute a current. Thus, the sudden collapse observed in all three strategies means that the network eventually fails to transport. The onset of this collapse is affected by the evolution strategy, in particular, the pseudo-Darwinian strategy leads to a nearly “optimized” network, i.e. a structure that carries most of the flux with minimal number of useless links (i.e. links that carry a negligible part of the flux). This can also be seen as an “economical” structure, where the transport function is kept with the least size of components and, hence, minimized maintenance cost^25^. However, going to an optimal system makes it fragile and dangerous because a small disturbance can lead to a sudden collapse. Therefore, for robustness of the network, a safety margin from the critical point $t_c$ should be considered, as known for human bronchial systems^5^.

When reaching $t_c$, the “optimized” structure becomes a chain connecting pre-selected source and drain nodes. While this structure is formally optimal, it is impractical for applications due to its “optimality” for the particular choice of the source and the drain. A much more challenging and practically relevant question would be the construction of the optimal structure for all (or for most) pairs of source and drain nodes. This problem will be investigated in a subsequent work.

This paper focused on the evolution of both transport and structural properties of scale-free networks under progressive link removal. Such transport-driven dynamics further extend common models of network evolutions^26–28^. In particular, our analysis helps to investigate the precursors of the transitions for links percolation applied to resistor networks^29^ or epidemic spreading^30^. More generally, this study can serve as a basis for illustrating generic evolution dynamics of complex networks governed by its transport properties. Different works already made the analogy between Kirchhoff’s laws and the conservation of mass equation, with applications to vehicular flow^31^, fractures in materials^32^, neuronal circuitry in the brain^33^. For instance, a direct analogy with random walks on graphs opens exciting perspectives for understanding diffusive transport and first-passage processes on evolving networks^34,35^. Our approach is also expected to stimulate further studies of link-based percolation in other networks such as in protein networks, where links can be lost with time (corresponding to the loss of some proteins’ functionalities), while proteins, the nodes of a network, remain present^36^.

## Supplementary information

Supplementary Information.

Supplementary Video

## Data Availability

Data sharing not applicable to this article as no datasets were generated or analysed during the current study.
