# Supplementary material for: Pseudo-Darwinian evolution of physical flows in complex networks
Source: Sci Rep. 2020 Sep 23;10:15477. doi: 10.1038/s41598-020-72379-8 (PMC7511406; doi:10.1038/s41598-020-72379-8)
Supplement: Supplementary file 1 — Supplementary Information. [file 41598_2020_72379_MOESM1_ESM.pdf]

Supplementary Material for the paper "Pseudo-Darwinian evolution of physical flows in complex networks"

Geoffroy Berthelot,[1,2,3] Liubov Tupikina,[4,5] Min-Yeong Kang,[6] Bernard Sapoval,[6] and Denis S. Grebenkov[6]

[1] *Centre de Mathématiques Appliquées, CNRS – Ecole Polytechnique, IP Paris, 91128 Palaiseau, France*
[2] *Research Laboratory for Interdisciplinary Studies (RELAIS), 75012 Paris, France*
[3] *Institut National du Sport, de l'Expertise et de la Performance (INSEP), 75012 Paris, France*
[4] *The Center for Research and Interdisciplinarity, Paris, France 75004*
[5] *Bell Labs Nokia, France*
[6] *Laboratoire de Physique de la Matière Condensée (UMR 7643), CNRS – Ecole Polytechnique, IP Paris, 91128 Palaiseau, France*

This Supplementary Material contains a link to the online video illustrating the Darwinian evolution of one random realization of the network with $N_0 = 30 \times 30$ nodes, $\gamma = 2.5$ and $\beta = 1$ (see also Fig. 4 in the main text).
